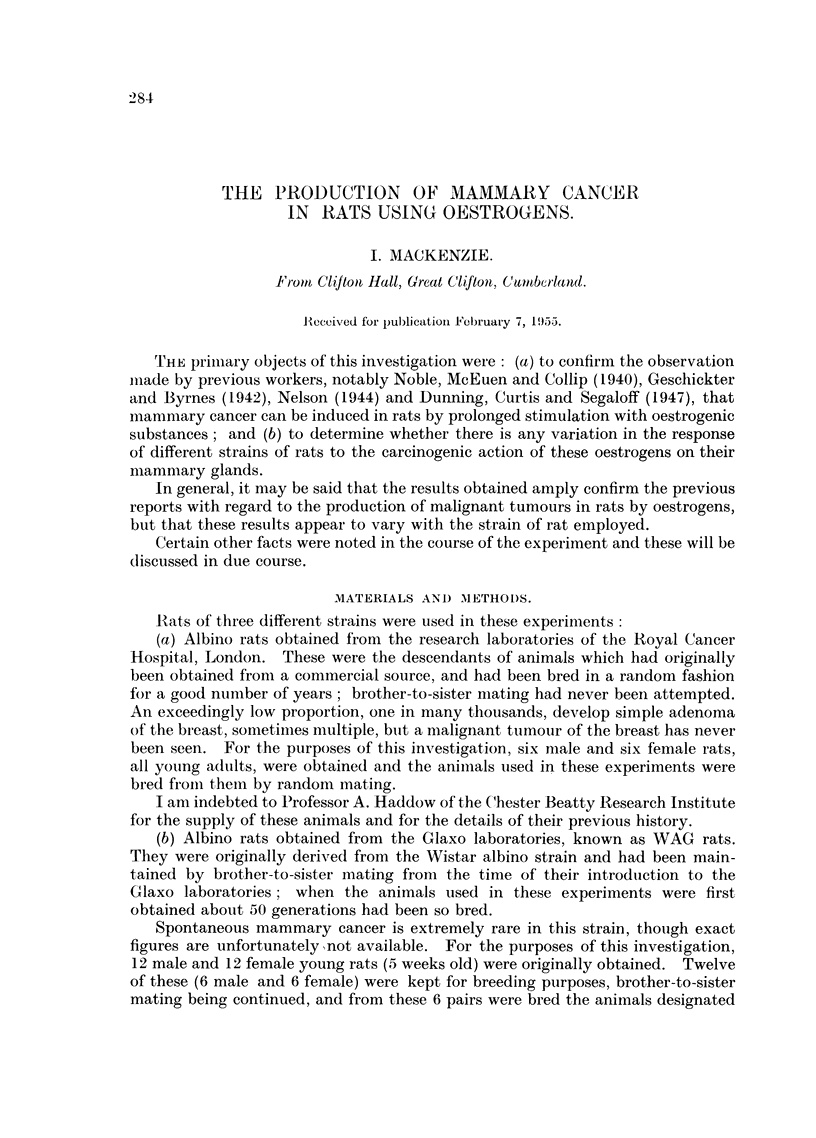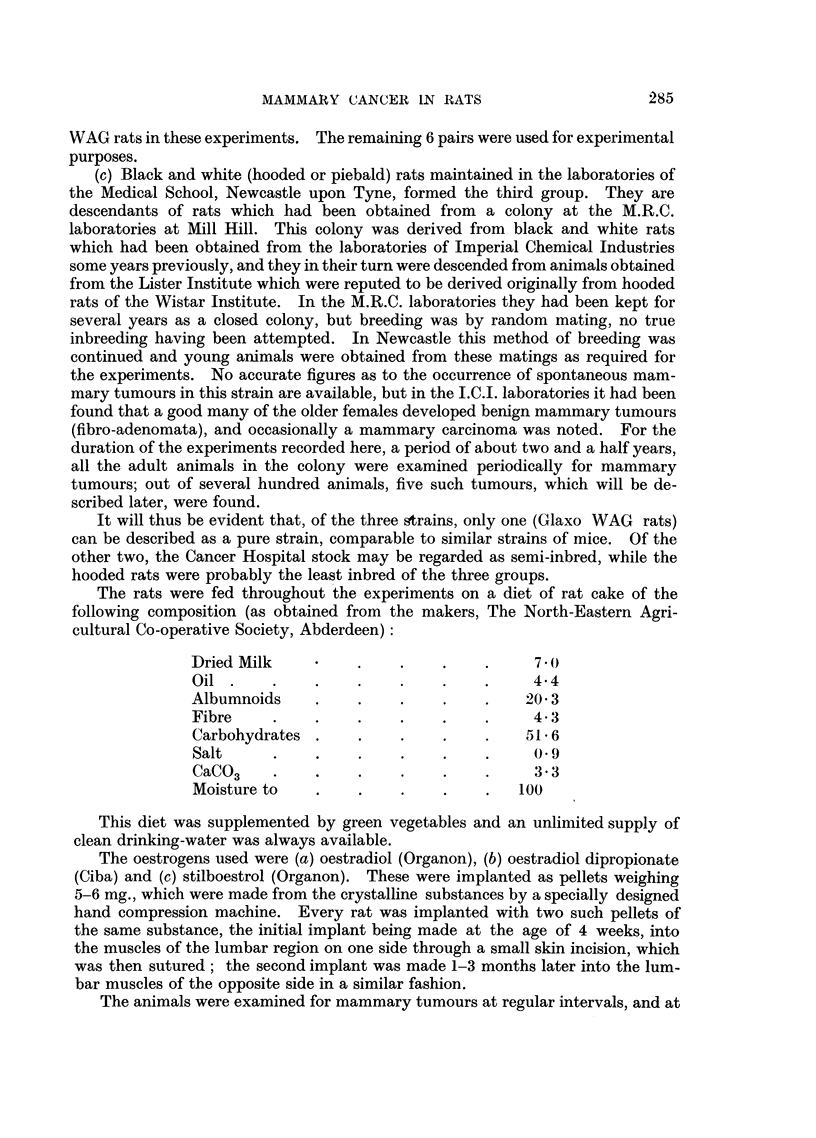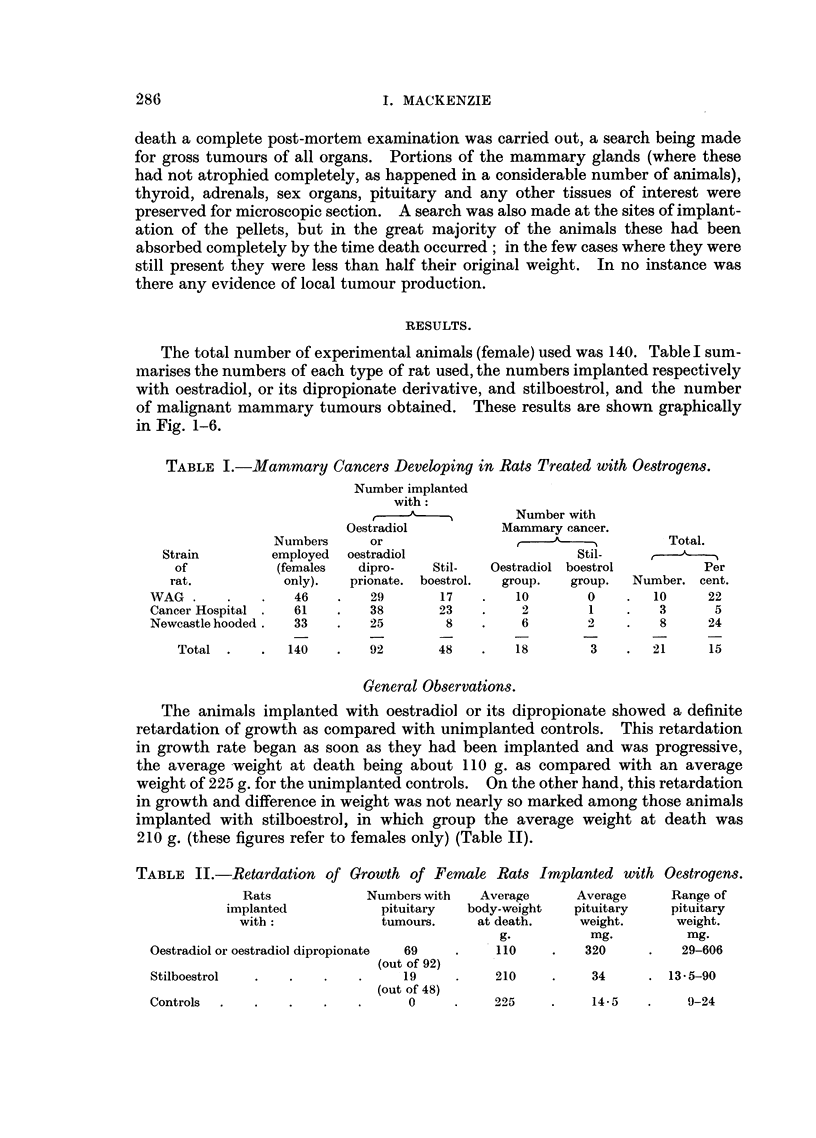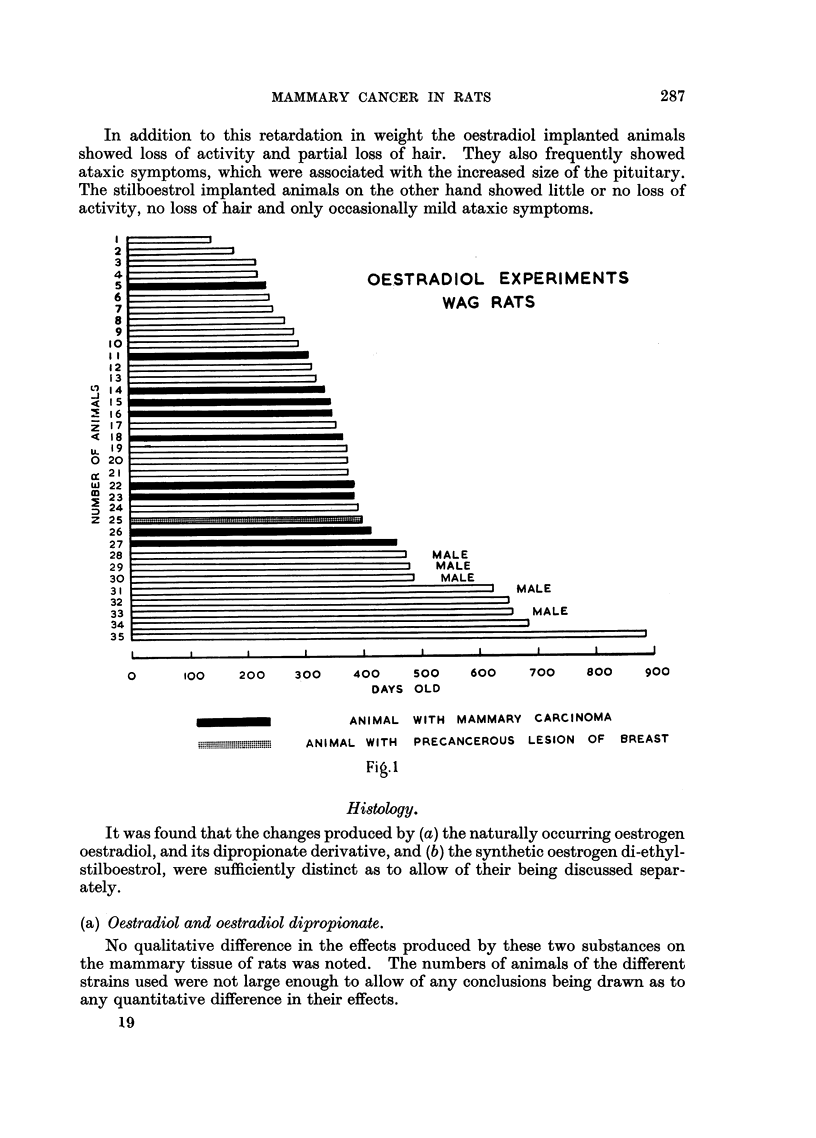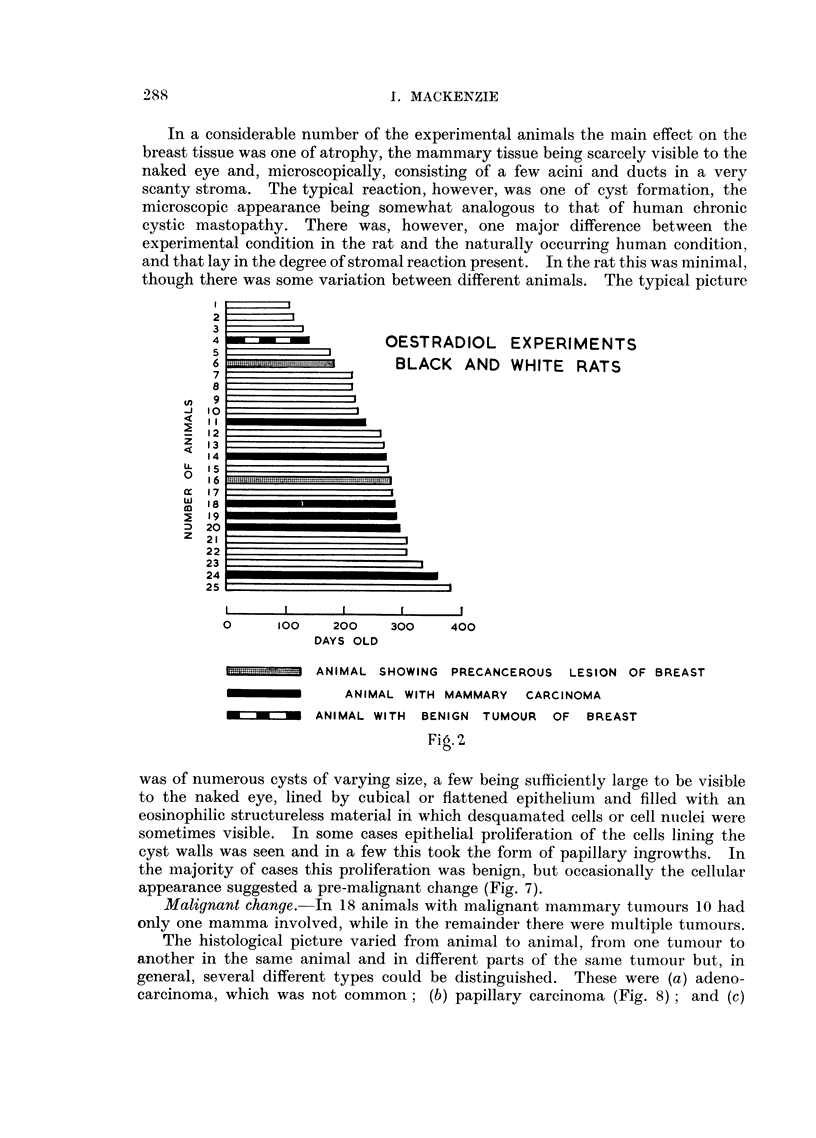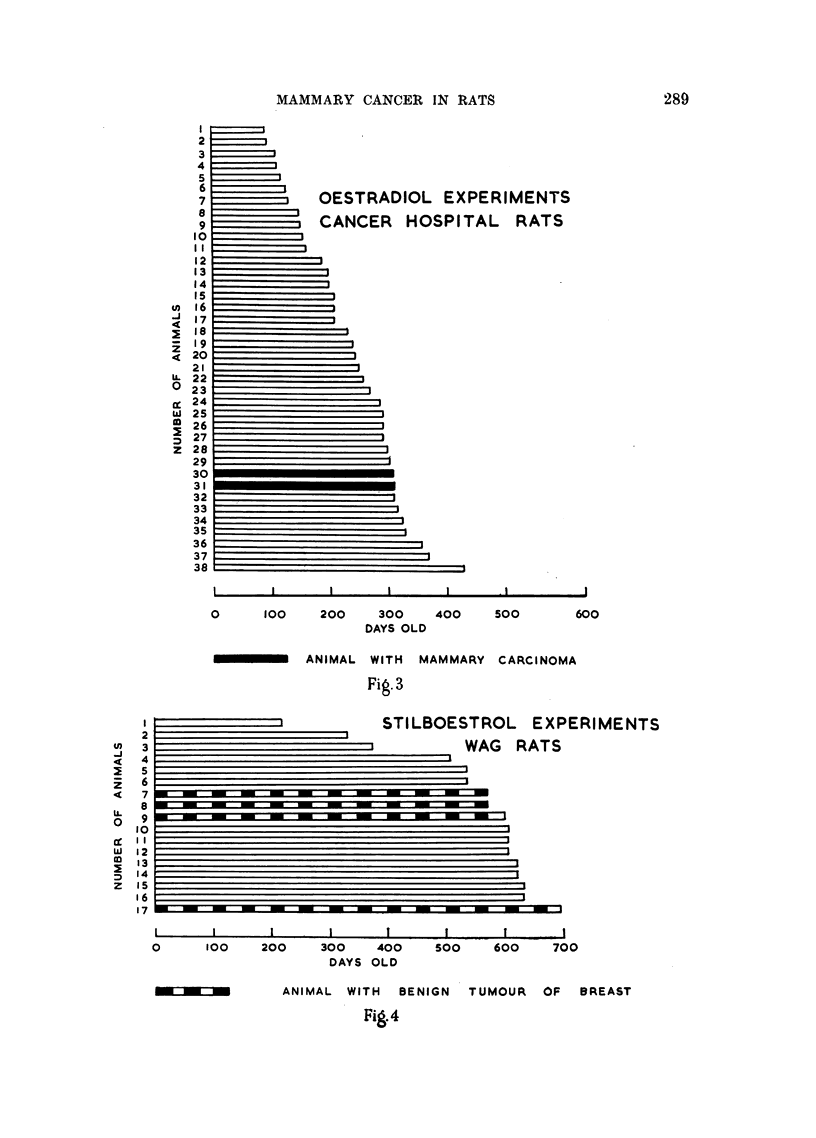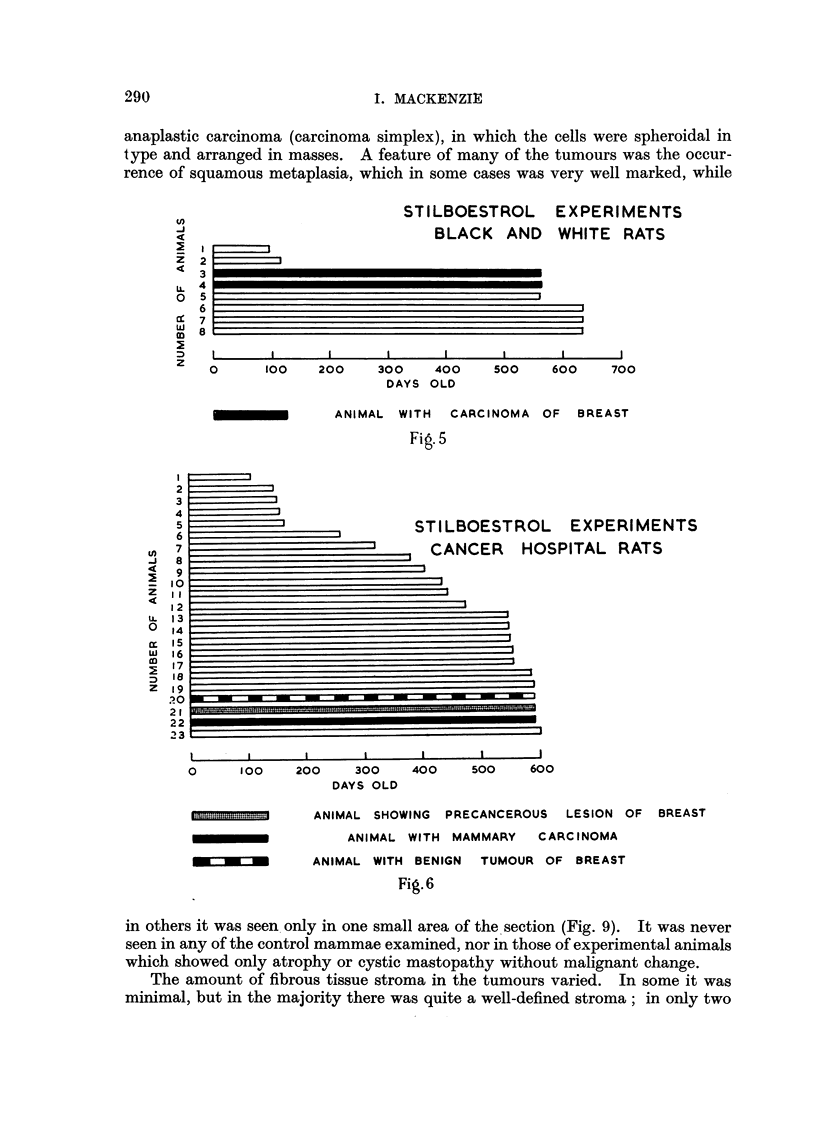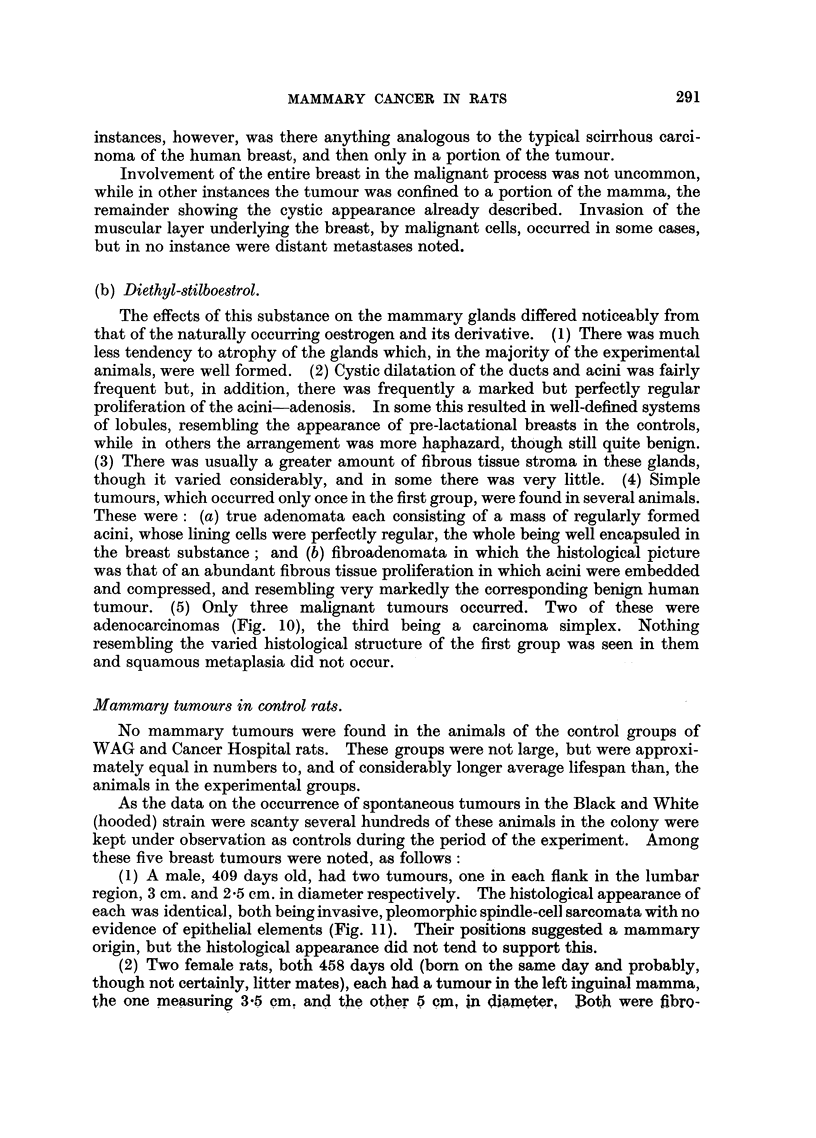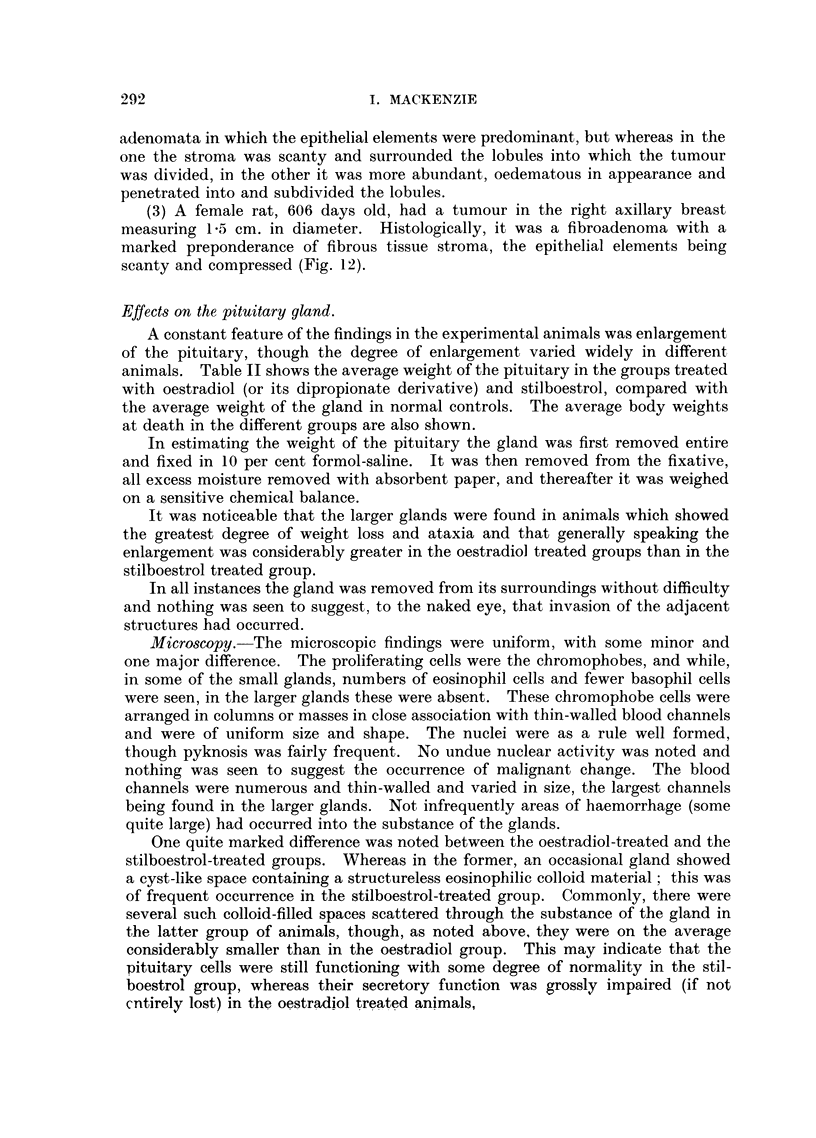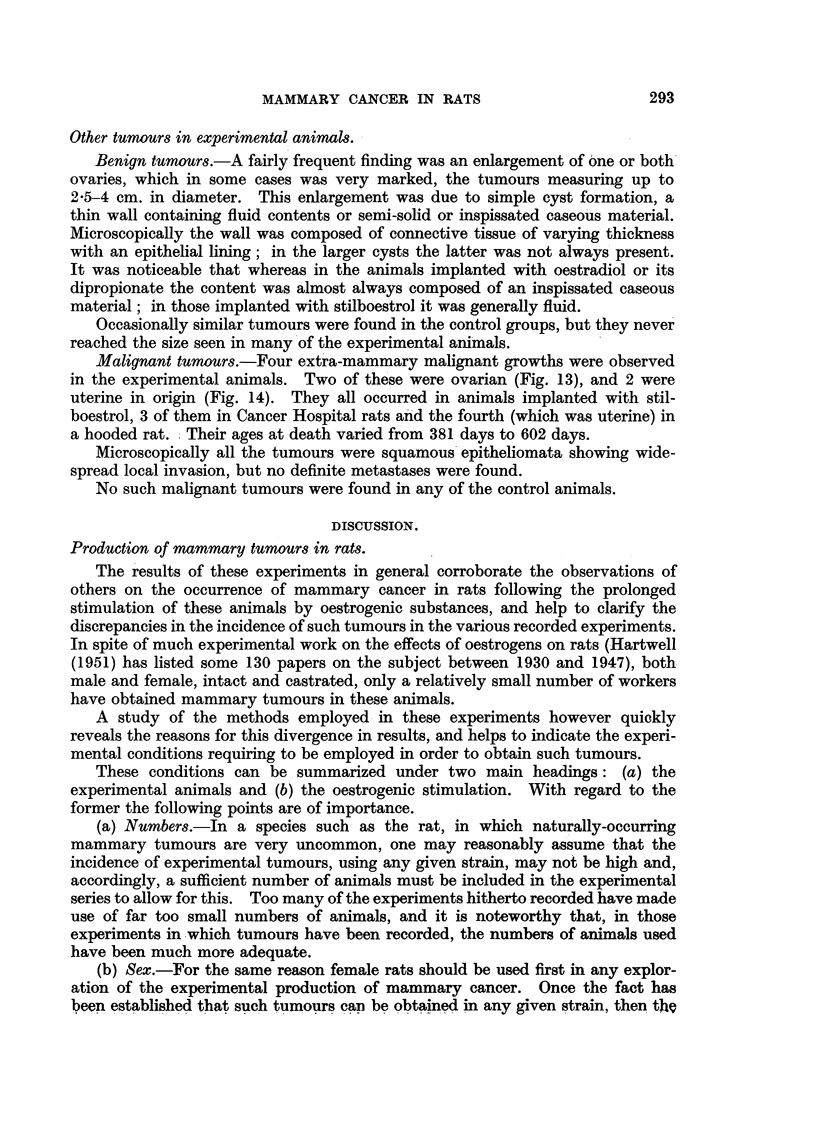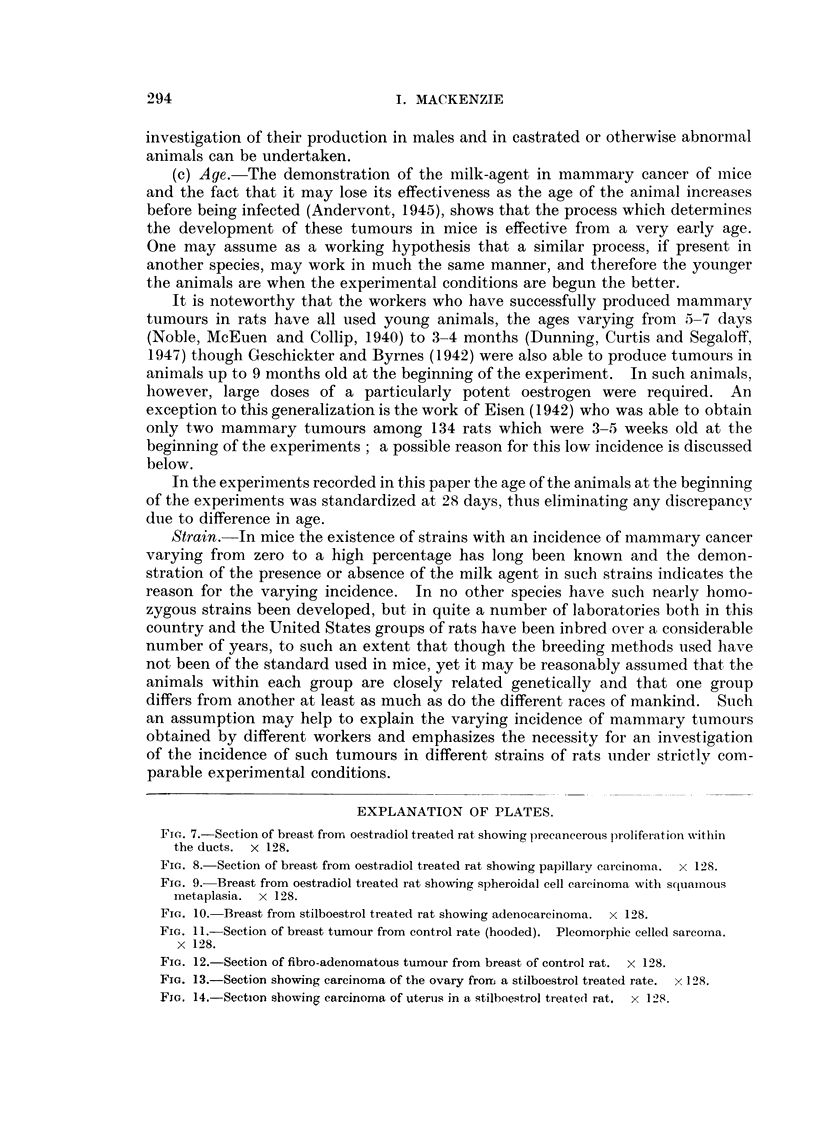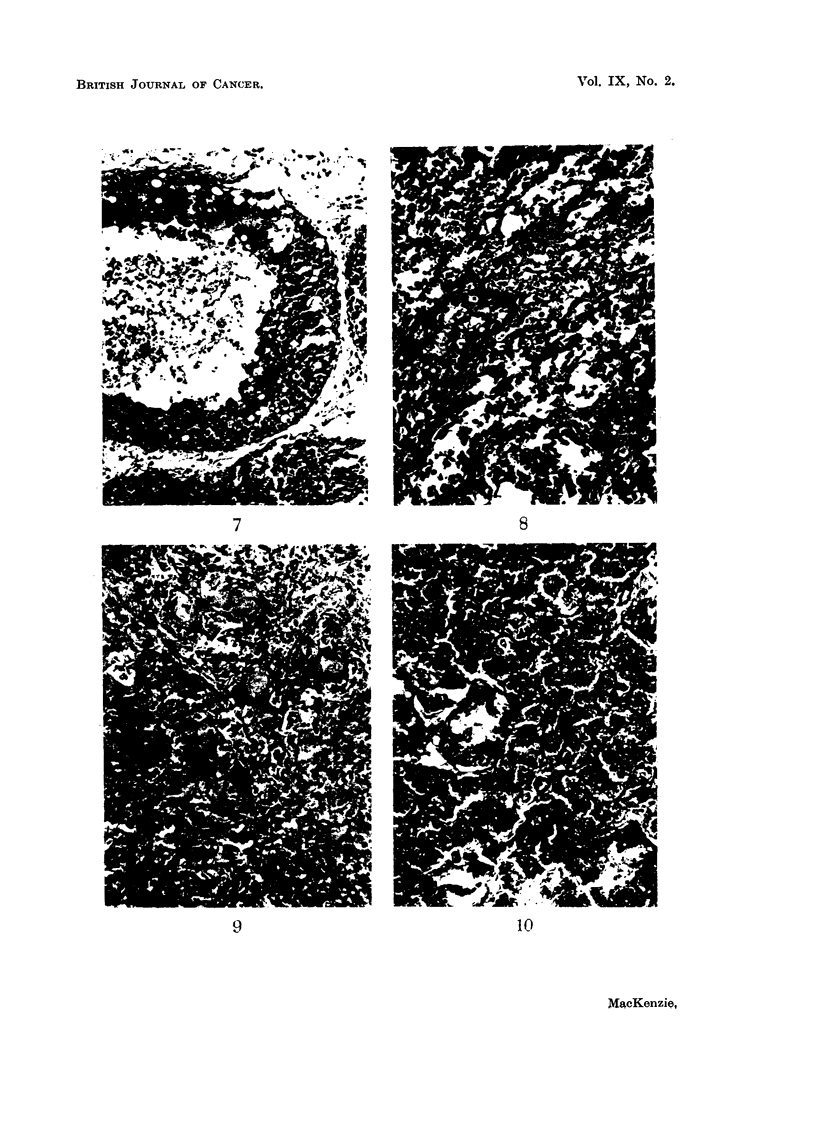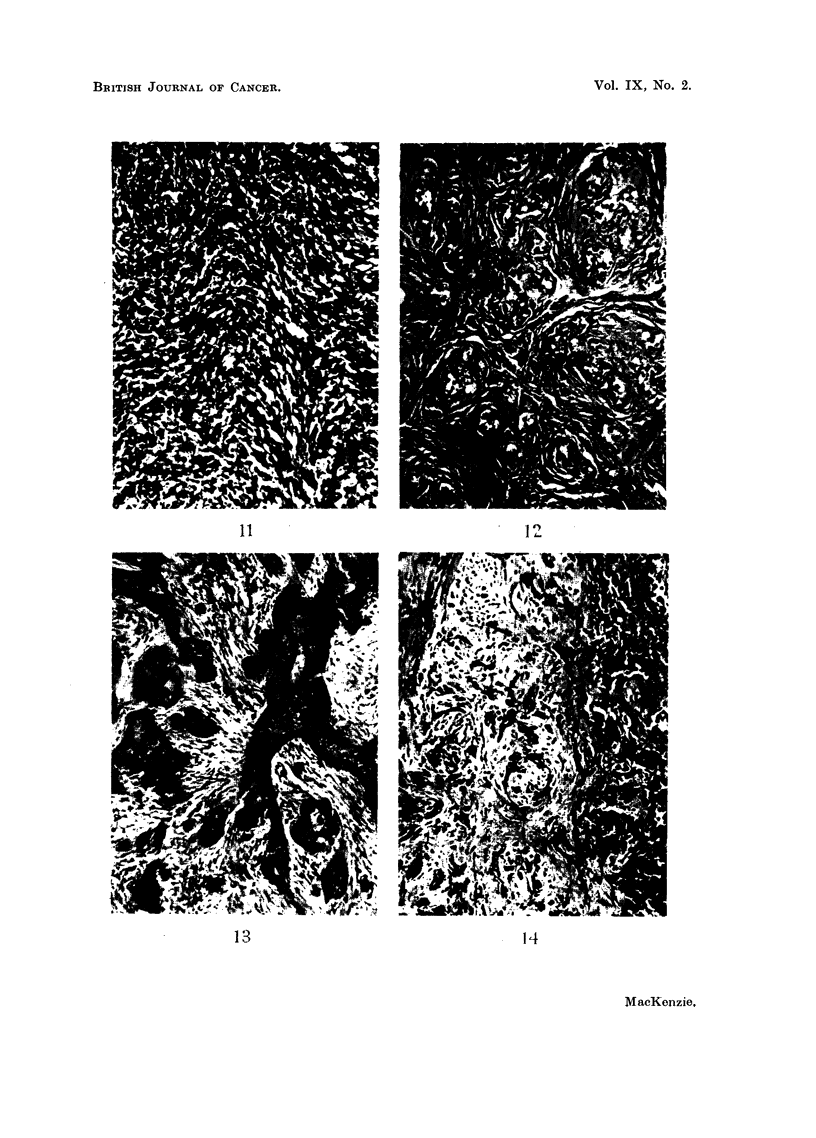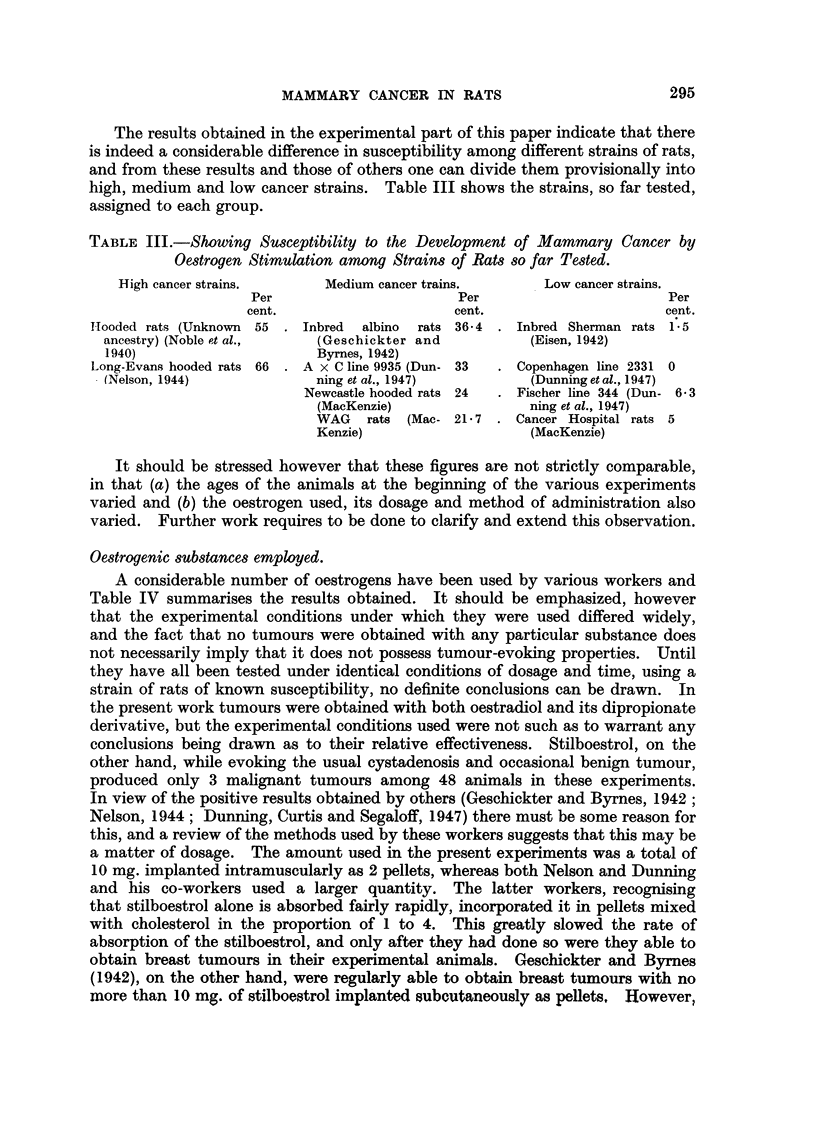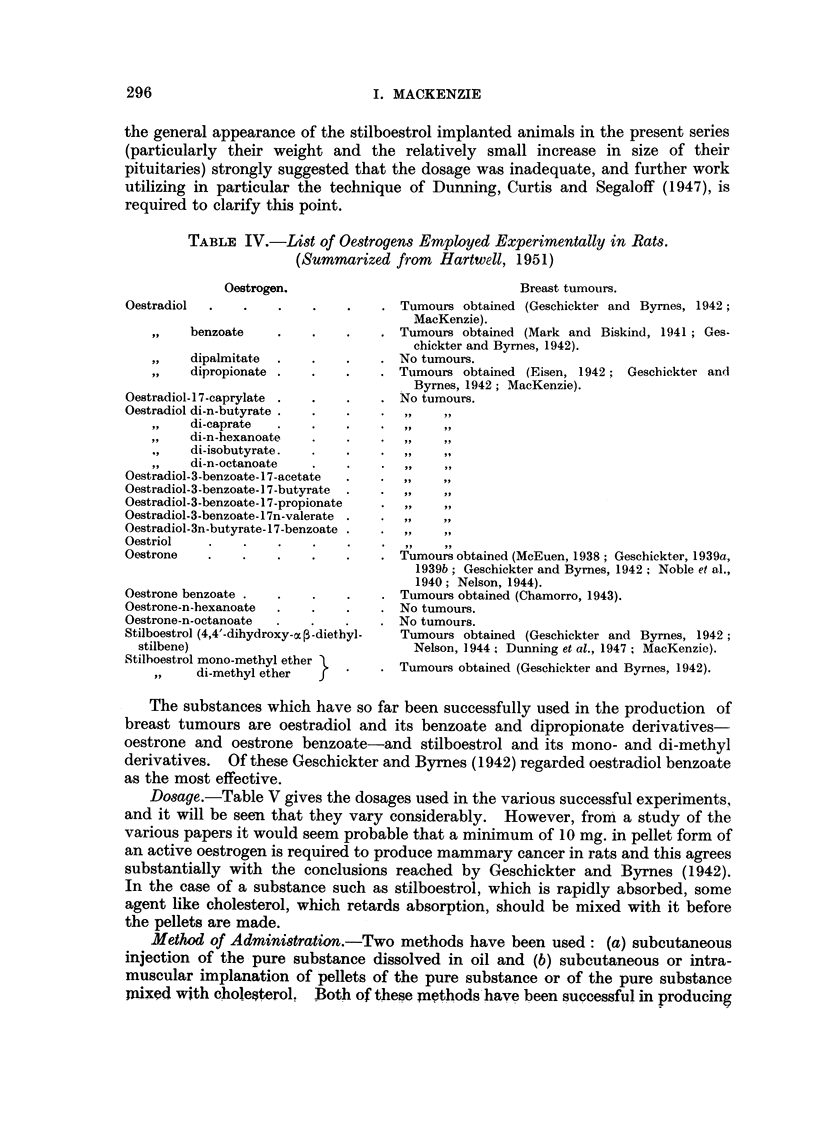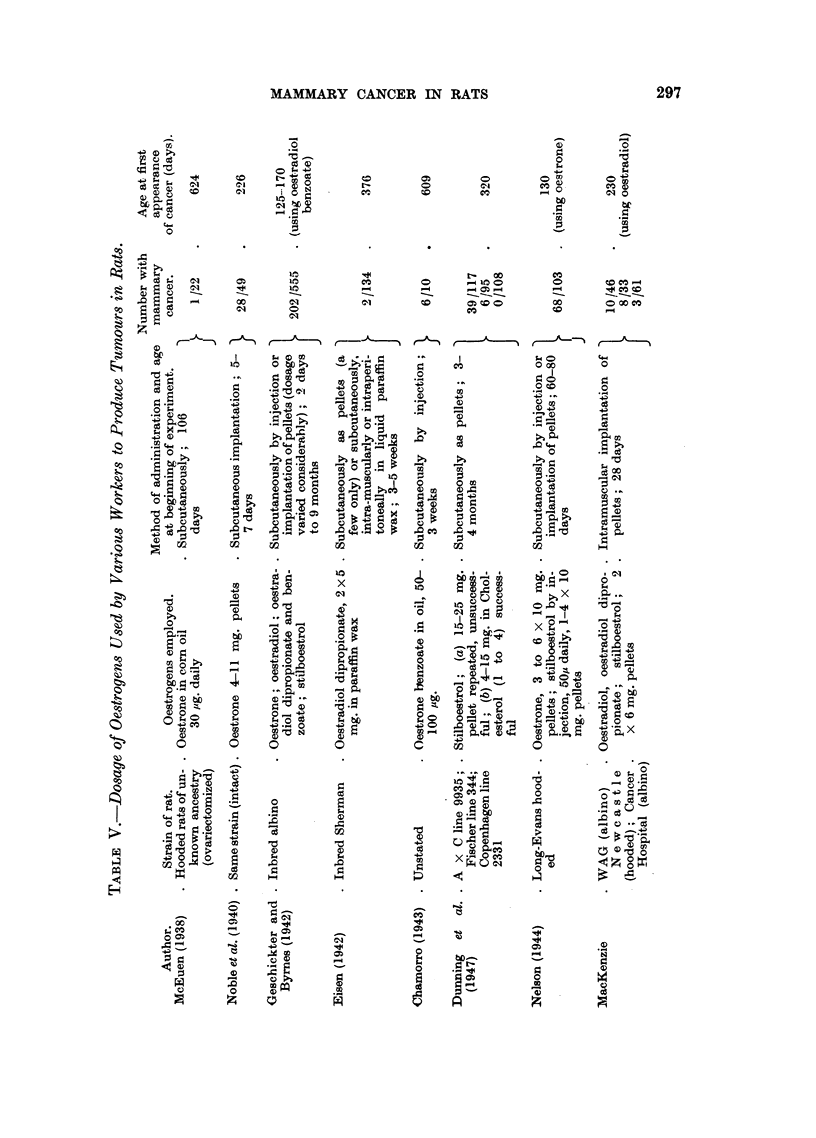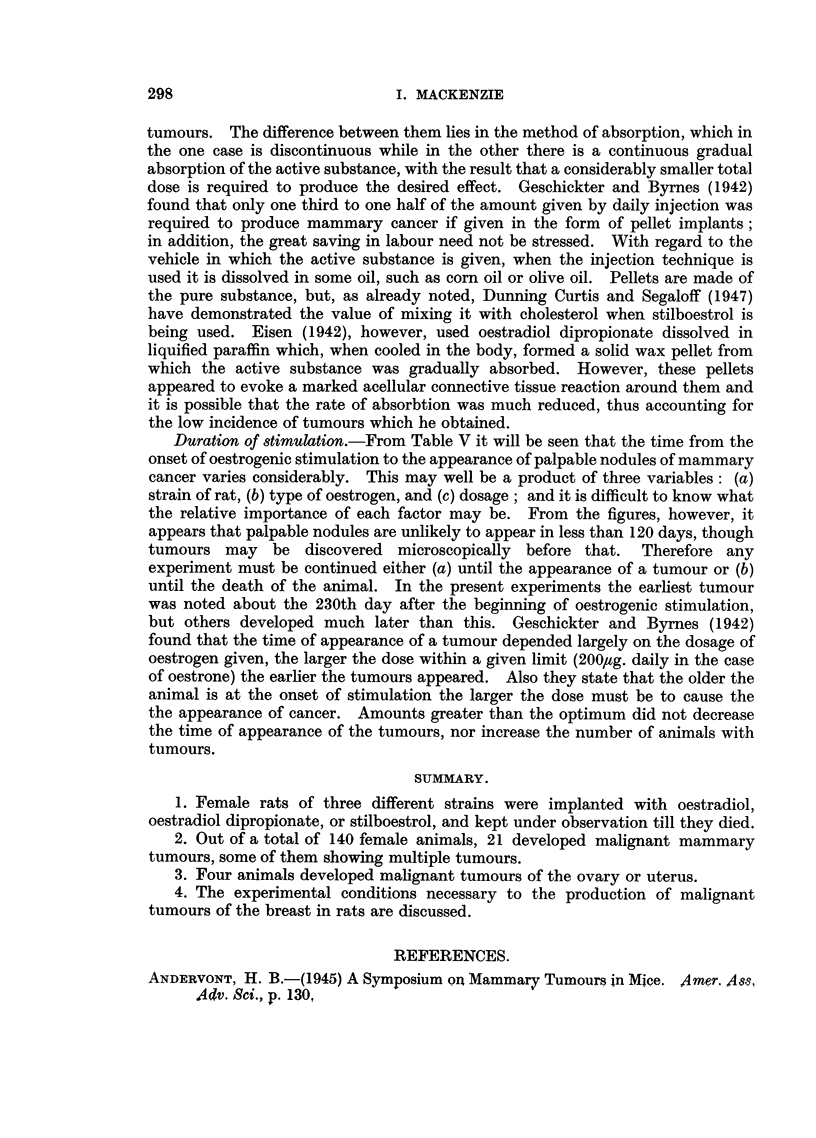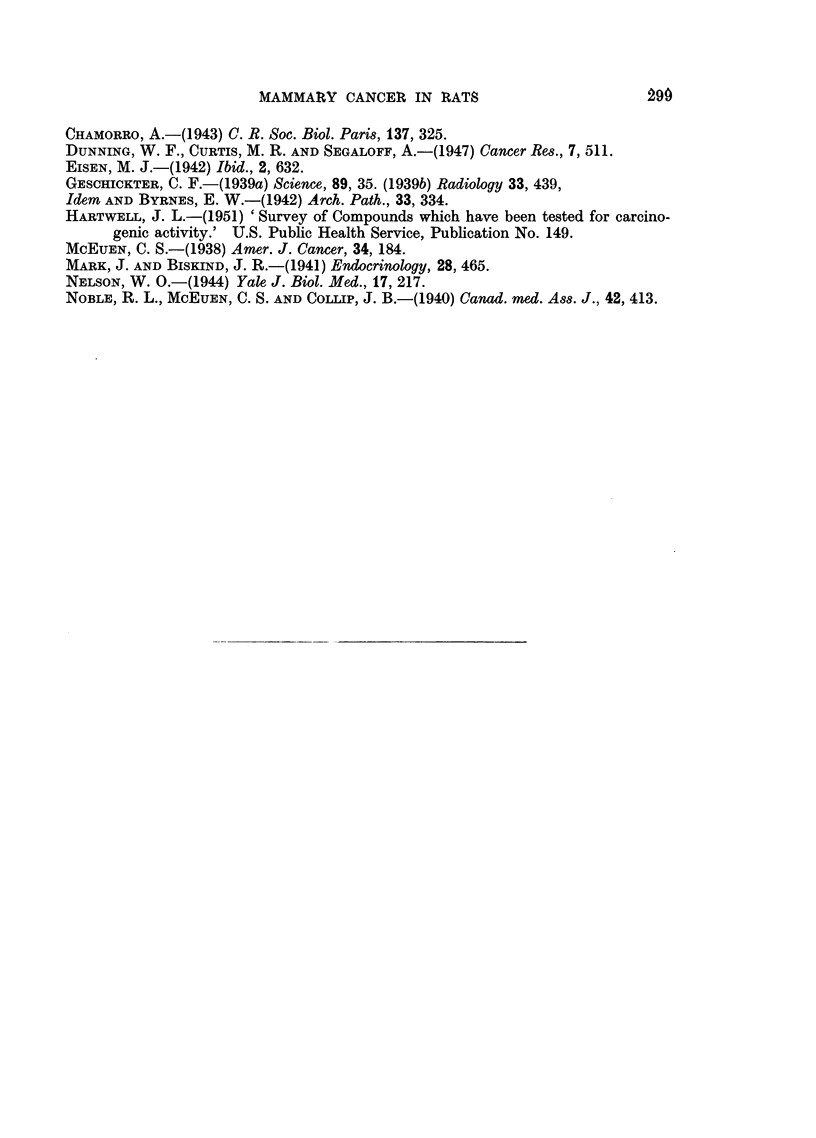# The Production of Mammary Cancer in Rats Using Oestrogens

**DOI:** 10.1038/bjc.1955.25

**Published:** 1955-06

**Authors:** I. MacKenzie

## Abstract

**Images:**


### THE PRODUCTION OF MAMMARY CANCER IN RATS USING OESTROGENS.

I. MACKENZIE.

*From Clifton Hall, Great Clifton, Cumberland.*

Received for publication February 7, 1955.

THE primary objects of this investigation were : (*a*) to confirm the observation made by previous workers, notably Noble, McEuen and Collip (1940), Geschickter and Byrnes (1942), Nelson (1944) and Dunning, Curtis and Segaloff (1947), that mammary cancer can be induced in rats by prolonged stimulation with oestrogenic substances ; and (*b*) to determine whether there is any variation in the response of different strains of rats to the carcinogenic action of these oestrogens on their mammary glands.

In general, it may be said that the results obtained amply confirm the previous reports with regard to the production of malignant tumours in rats by oestrogens, but that these results appear to vary with the strain of rat employed.

Certain other facts were noted in the course of the experiment and these will be discussed in due course.

### MATERIALS AND METHODS.

Rats of three different strains were used in these experiments :

(*a*) Albino rats obtained from the research laboratories of the Royal Cancer Hospital, London. These were the descendants of animals which had originally been obtained from a commercial source, and had been bred in a random fashion for a good number of years ; brother-to-sister mating had never been attempted. An exceedingly low proportion, one in many thousands, develop simple adenoma of the breast, sometimes multiple, but a malignant tumour of the breast has never been seen. For the purposes of this investigation, six male and six female rats, all young adults, were obtained and the animals used in these experiments were bred from them by random mating.

I am indebted to Professor A. Haddow of the Chester Beatty Research Institute for the supply of these animals and for the details of their previous history.

(*b*) Albino rats obtained from the Glaxo laboratories, known as WAG rats. They were originally derived from the Wistar albino strain and had been maintained by brother-to-sister mating from the time of their introduction to the Glaxo laboratories ; when the animals used in these experiments were first obtained about 50 generations had been so bred.

Spontaneous mammary cancer is extremely rare in this strain, though exact figures are unfortunately not available. For the purposes of this investigation, 12 male and 12 female young rats (5 weeks old) were originally obtained. Twelve of these (6 male and 6 female) were kept for breeding purposes, brother-to-sister mating being continued, and from these 6 pairs were bred the animals designated

WAG rats in these experiments. The remaining 6 pairs were used for experimental purposes.

(c) Black and white (hooded or piebald) rats maintained in the laboratories of the Medical School, Newcastle upon Tyne, formed the third group. They are descendants of rats which had been obtained from a colony at the M.R.C. laboratories at Mill Hill. This colony was derived from black and white rats which had been obtained from the laboratories of Imperial Chemical Industries some years previously, and they in their turn were descended from animals obtained from the Lister Institute which were reputed to be derived originally from hooded rats of the Wistar Institute. In the M.R.C. laboratories they had been kept for several years as a closed colony, but breeding was by random mating, no true inbreeding having been attempted. In Newcastle this method of breeding was continued and young animals were obtained from these matings as required for the experiments. No accurate figures as to the occurrence of spontaneous mammary tumours in this strain are available, but in the I.C.I. laboratories it had been found that a good many of the older females developed benign mammary tumours (fibro-adenomata), and occasionally a mammary carcinoma was noted. For the duration of the experiments recorded here, a period of about two and a half years, all the adult animals in the colony were examined periodically for mammary tumours; out of several hundred animals, five such tumours, which will be described later, were found.

It will thus be evident that, of the three strains, only one (Glaxo WAG rats) can be described as a pure strain, comparable to similar strains of mice. Of the other two, the Cancer Hospital stock may be regarded as semi-inbred, while the hooded rats were probably the least inbred of the three groups.

The rats were fed throughout the experiments on a diet of rat cake of the following composition (as obtained from the makers, The North-Eastern Agricultural Co-operative Society, Abderdeen):

| | |
|---|---|
| Dried Milk . . . . . . | 7·0 |
| Oil . . . . . . . | 4·4 |
| Albumnoids . . . . . . | 20·3 |
| Fibre . . . . . . | 4·3 |
| Carbohydrates . . . . . . | 51·6 |
| Salt . . . . . . | 0·9 |
| $CaCO_3$ . . . . . . | 3·3 |
| Moisture to . . . . . . | 100 |

This diet was supplemented by green vegetables and an unlimited supply of clean drinking-water was always available.

The oestrogens used were (a) oestradiol (Organon), (b) oestradiol dipropionate (Ciba) and (c) stilboestrol (Organon). These were implanted as pellets weighing 5–6 mg., which were made from the crystalline substances by a specially designed hand compression machine. Every rat was implanted with two such pellets of the same substance, the initial implant being made at the age of 4 weeks, into the muscles of the lumbar region on one side through a small skin incision, which was then sutured; the second implant was made 1–3 months later into the lumbar muscles of the opposite side in a similar fashion.

The animals were examined for mammary tumours at regular intervals, and at death a complete post-mortem examination was carried out, a search being made for gross tumours of all organs. Portions of the mammary glands (where these had not atrophied completely, as happened in a considerable number of animals), thyroid, adrenals, sex organs, pituitary and any other tissues of interest were preserved for microscopic section. A search was also made at the sites of implantation of the pellets, but in the great majority of the animals these had been absorbed completely by the time death occurred ; in the few cases where they were still present they were less than half their original weight. In no instance was there any evidence of local tumour production.

### RESULTS.

The total number of experimental animals (female) used was 140. Table I summarises the numbers of each type of rat used, the numbers implanted respectively with oestradiol, or its dipropionate derivative, and stilboestrol, and the number of malignant mammary tumours obtained. These results are shown graphically in Fig. 1–6.

TABLE I.—*Mammary Cancers Developing in Rats Treated with Oestrogens.*

| Strain of rat. | Numbers employed (females only). | Number implanted with: Oestradiol or oestradiol dipropionate. | Stilboestrol. | Number with Mammary cancer. Oestradiol group. | Stilboestrol group. | Total. Number. | Per cent. |
|---|---|---|---|---|---|---|---|
| WAG | 46 | 29 | 17 | 10 | 0 | 10 | 22 |
| Cancer Hospital | 61 | 38 | 23 | 2 | 1 | 3 | 5 |
| Newcastle hooded | 33 | 25 | 8 | 6 | 2 | 8 | 24 |
| Total | 140 | 92 | 48 | 18 | 3 | 21 | 15 |

### *General Observations.*

The animals implanted with oestradiol or its dipropionate showed a definite retardation of growth as compared with unimplanted controls. This retardation in growth rate began as soon as they had been implanted and was progressive, the average weight at death being about 110 g. as compared with an average weight of 225 g. for the unimplanted controls. On the other hand, this retardation in growth and difference in weight was not nearly so marked among those animals implanted with stilboestrol, in which group the average weight at death was 210 g. (these figures refer to females only) (Table II).

TABLE II.—*Retardation of Growth of Female Rats Implanted with Oestrogens.*

| Rats implanted with: | Numbers with pituitary tumours. | Average body-weight at death. g. | Average pituitary weight. mg. | Range of pituitary weight. mg. |
|---|---|---|---|---|
| Oestradiol or oestradiol dipropionate | 69 (out of 92) | 110 | 320 | 29–606 |
| Stilboestrol | 19 (out of 48) | 210 | 34 | 13·5–90 |
| Controls | 0 | 225 | 14·5 | 9–24 |

In addition to this retardation in weight the oestradiol implanted animals showed loss of activity and partial loss of hair. They also frequently showed ataxic symptoms, which were associated with the increased size of the pituitary. The stilboestrol implanted animals on the other hand showed little or no loss of activity, no loss of hair and only occasionally mild ataxic symptoms.

Fig.1

*Histology.*

It was found that the changes produced by (*a*) the naturally occurring oestrogen oestradiol, and its dipropionate derivative, and (*b*) the synthetic oestrogen di-ethyl-stilboestrol, were sufficiently distinct as to allow of their being discussed separately.

(a) *Oestradiol and oestradiol dipropionate.*

No qualitative difference in the effects produced by these two substances on the mammary tissue of rats was noted. The numbers of animals of the different strains used were not large enough to allow of any conclusions being drawn as to any quantitative difference in their effects.

In a considerable number of the experimental animals the main effect on the breast tissue was one of atrophy, the mammary tissue being scarcely visible to the naked eye and, microscopically, consisting of a few acini and ducts in a very scanty stroma. The typical reaction, however, was one of cyst formation, the microscopic appearance being somewhat analogous to that of human chronic cystic mastopathy. There was, however, one major difference between the experimental condition in the rat and the naturally occurring human condition, and that lay in the degree of stromal reaction present. In the rat this was minimal, though there was some variation between different animals. The typical picture

Fig. 2

was of numerous cysts of varying size, a few being sufficiently large to be visible to the naked eye, lined by cubical or flattened epithelium and filled with an eosinophilic structureless material in which desquamated cells or cell nuclei were sometimes visible. In some cases epithelial proliferation of the cells lining the cyst walls was seen and in a few this took the form of papillary ingrowths. In the majority of cases this proliferation was benign, but occasionally the cellular appearance suggested a pre-malignant change (Fig. 7).

*Malignant change.*—In 18 animals with malignant mammary tumours 10 had only one mamma involved, while in the remainder there were multiple tumours.

The histological picture varied from animal to animal, from one tumour to another in the same animal and in different parts of the same tumour but, in general, several different types could be distinguished. These were (*a*) adeno-carcinoma, which was not common ; (*b*) papillary carcinoma (Fig. 8) ; and (*c*)

OESTRADIOL EXPERIMENTS
CANCER HOSPITAL RATS
NUMBER OF ANIMALS
1
2
3
4
5
6
7
8
9
10
11
12
13
14
15
16
17
18
19
20
21
22
23
24
25
26
27
28
29
30
31
32
33
34
35
36
37
38
0
100
200
300
400
500
600
DAYS OLD
ANIMAL WITH MAMMARY CARCINOMA

Fig. 3

STILBOESTROL EXPERIMENTS

WAG RATS

NUMBER OF ANIMALS

1

2

3

4

5

6

7

8

9

10

11

12

13

14

15

16

17

0

100

200

300

400

500

600

700

DAYS OLD

ANIMAL WITH BENIGN TUMOUR OF BREAST

Fig. 4

anaplastic carcinoma (carcinoma simplex), in which the cells were spheroidal in type and arranged in masses. A feature of many of the tumours was the occurrence of squamous metaplasia, which in some cases was very well marked, while

Fig. 5

Fig. 6

in others it was seen only in one small area of the section (Fig. 9). It was never seen in any of the control mammae examined, nor in those of experimental animals which showed only atrophy or cystic mastopathy without malignant change.

The amount of fibrous tissue stroma in the tumours varied. In some it was minimal, but in the majority there was quite a well-defined stroma ; in only two instances, however, was there anything analogous to the typical scirrhous carcinoma of the human breast, and then only in a portion of the tumour.

Involvement of the entire breast in the malignant process was not uncommon, while in other instances the tumour was confined to a portion of the mamma, the remainder showing the cystic appearance already described. Invasion of the muscular layer underlying the breast, by malignant cells, occurred in some cases, but in no instance were distant metastases noted.

(b) *Diethyl-stilboestrol.*

The effects of this substance on the mammary glands differed noticeably from that of the naturally occurring oestrogen and its derivative. (1) There was much less tendency to atrophy of the glands which, in the majority of the experimental animals, were well formed. (2) Cystic dilatation of the ducts and acini was fairly frequent but, in addition, there was frequently a marked but perfectly regular proliferation of the acini—adenosis. In some this resulted in well-defined systems of lobules, resembling the appearance of pre-lactational breasts in the controls, while in others the arrangement was more haphazard, though still quite benign. (3) There was usually a greater amount of fibrous tissue stroma in these glands, though it varied considerably, and in some there was very little. (4) Simple tumours, which occurred only once in the first group, were found in several animals. These were : (*a*) true adenomata each consisting of a mass of regularly formed acini, whose lining cells were perfectly regular, the whole being well encapsuled in the breast substance ; and (*b*) fibroadenomata in which the histological picture was that of an abundant fibrous tissue proliferation in which acini were embedded and compressed, and resembling very markedly the corresponding benign human tumour. (5) Only three malignant tumours occurred. Two of these were adenocarcinomas (Fig. 10), the third being a carcinoma simplex. Nothing resembling the varied histological structure of the first group was seen in them and squamous metaplasia did not occur.

*Mammary tumours in control rats.*

No mammary tumours were found in the animals of the control groups of WAG and Cancer Hospital rats. These groups were not large, but were approximately equal in numbers to, and of considerably longer average lifespan than, the animals in the experimental groups.

As the data on the occurrence of spontaneous tumours in the Black and White (hooded) strain were scanty several hundreds of these animals in the colony were kept under observation as controls during the period of the experiment. Among these five breast tumours were noted, as follows :

(1) A male, 409 days old, had two tumours, one in each flank in the lumbar region, 3 cm. and 2·5 cm. in diameter respectively. The histological appearance of each was identical, both being invasive, pleomorphic spindle-cell sarcomata with no evidence of epithelial elements (Fig. 11). Their positions suggested a mammary origin, but the histological appearance did not tend to support this.

(2) Two female rats, both 458 days old (born on the same day and probably, though not certainly, litter mates), each had a tumour in the left inguinal mamma, the one measuring 3·5 cm. and the other 5 cm. in diameter. Both were fibroadenomata in which the epithelial elements were predominant, but whereas in the one the stroma was scanty and surrounded the lobules into which the tumour was divided, in the other it was more abundant, oedematous in appearance and penetrated into and subdivided the lobules.

(3) A female rat, 606 days old, had a tumour in the right axillary breast measuring 1·5 cm. in diameter. Histologically, it was a fibroadenoma with a marked preponderance of fibrous tissue stroma, the epithelial elements being scanty and compressed (Fig. 12).

*Effects on the pituitary gland.*

A constant feature of the findings in the experimental animals was enlargement of the pituitary, though the degree of enlargement varied widely in different animals. Table II shows the average weight of the pituitary in the groups treated with oestradiol (or its dipropionate derivative) and stilboestrol, compared with the average weight of the gland in normal controls. The average body weights at death in the different groups are also shown.

In estimating the weight of the pituitary the gland was first removed entire and fixed in 10 per cent formol-saline. It was then removed from the fixative, all excess moisture removed with absorbent paper, and thereafter it was weighed on a sensitive chemical balance.

It was noticeable that the larger glands were found in animals which showed the greatest degree of weight loss and ataxia and that generally speaking the enlargement was considerably greater in the oestradiol treated groups than in the stilboestrol treated group.

In all instances the gland was removed from its surroundings without difficulty and nothing was seen to suggest, to the naked eye, that invasion of the adjacent structures had occurred.

*Microscopy.*—The microscopic findings were uniform, with some minor and one major difference. The proliferating cells were the chromophobes, and while, in some of the small glands, numbers of eosinophil cells and fewer basophil cells were seen, in the larger glands these were absent. These chromophobe cells were arranged in columns or masses in close association with thin-walled blood channels and were of uniform size and shape. The nuclei were as a rule well formed, though pyknosis was fairly frequent. No undue nuclear activity was noted and nothing was seen to suggest the occurrence of malignant change. The blood channels were numerous and thin-walled and varied in size, the largest channels being found in the larger glands. Not infrequently areas of haemorrhage (some quite large) had occurred into the substance of the glands.

One quite marked difference was noted between the oestradiol-treated and the stilboestrol-treated groups. Whereas in the former, an occasional gland showed a cyst-like space containing a structureless eosinophilic colloid material ; this was of frequent occurrence in the stilboestrol-treated group. Commonly, there were several such colloid-filled spaces scattered through the substance of the gland in the latter group of animals, though, as noted above, they were on the average considerably smaller than in the oestradiol group. This may indicate that the pituitary cells were still functioning with some degree of normality in the stilboestrol group, whereas their secretory function was grossly impaired (if not entirely lost) in the oestradiol treated animals,

*Other tumours in experimental animals.*

*Benign tumours.*—A fairly frequent finding was an enlargement of one or both ovaries, which in some cases was very marked, the tumours measuring up to 2·5–4 cm. in diameter. This enlargement was due to simple cyst formation, a thin wall containing fluid contents or semi-solid or inspissated caseous material. Microscopically the wall was composed of connective tissue of varying thickness with an epithelial lining ; in the larger cysts the latter was not always present. It was noticeable that whereas in the animals implanted with oestradiol or its dipropionate the content was almost always composed of an inspissated caseous material ; in those implanted with stilboestrol it was generally fluid.

Occasionally similar tumours were found in the control groups, but they never reached the size seen in many of the experimental animals.

*Malignant tumours.*—Four extra-mammary malignant growths were observed in the experimental animals. Two of these were ovarian (Fig. 13), and 2 were uterine in origin (Fig. 14). They all occurred in animals implanted with stilboestrol, 3 of them in Cancer Hospital rats and the fourth (which was uterine) in a hooded rat. Their ages at death varied from 381 days to 602 days.

Microscopically all the tumours were squamous epitheliomata showing widespread local invasion, but no definite metastases were found.

No such malignant tumours were found in any of the control animals.

### DISCUSSION.

*Production of mammary tumours in rats.*

The results of these experiments in general corroborate the observations of others on the occurrence of mammary cancer in rats following the prolonged stimulation of these animals by oestrogenic substances, and help to clarify the discrepancies in the incidence of such tumours in the various recorded experiments. In spite of much experimental work on the effects of oestrogens on rats (Hartwell (1951) has listed some 130 papers on the subject between 1930 and 1947), both male and female, intact and castrated, only a relatively small number of workers have obtained mammary tumours in these animals.

A study of the methods employed in these experiments however quickly reveals the reasons for this divergence in results, and helps to indicate the experimental conditions requiring to be employed in order to obtain such tumours.

These conditions can be summarized under two main headings : (*a*) the experimental animals and (*b*) the oestrogenic stimulation. With regard to the former the following points are of importance.

(a) *Numbers.*—In a species such as the rat, in which naturally-occurring mammary tumours are very uncommon, one may reasonably assume that the incidence of experimental tumours, using any given strain, may not be high and, accordingly, a sufficient number of animals must be included in the experimental series to allow for this. Too many of the experiments hitherto recorded have made use of far too small numbers of animals, and it is noteworthy that, in those experiments in which tumours have been recorded, the numbers of animals used have been much more adequate.

(b) *Sex.*—For the same reason female rats should be used first in any exploration of the experimental production of mammary cancer. Once the fact has been established that such tumours can be obtained in any given strain, then the investigation of their production in males and in castrated or otherwise abnormal animals can be undertaken.

(c) *Age*.—The demonstration of the milk-agent in mammary cancer of mice and the fact that it may lose its effectiveness as the age of the animal increases before being infected (Andervont, 1945), shows that the process which determines the development of these tumours in mice is effective from a very early age. One may assume as a working hypothesis that a similar process, if present in another species, may work in much the same manner, and therefore the younger the animals are when the experimental conditions are begun the better.

It is noteworthy that the workers who have successfully produced mammary tumours in rats have all used young animals, the ages varying from 5–7 days (Noble, McEuen and Collip, 1940) to 3–4 months (Dunning, Curtis and Segaloff, 1947) though Geschickter and Byrnes (1942) were also able to produce tumours in animals up to 9 months old at the beginning of the experiment. In such animals, however, large doses of a particularly potent oestrogen were required. An exception to this generalization is the work of Eisen (1942) who was able to obtain only two mammary tumours among 134 rats which were 3–5 weeks old at the beginning of the experiments ; a possible reason for this low incidence is discussed below.

In the experiments recorded in this paper the age of the animals at the beginning of the experiments was standardized at 28 days, thus eliminating any discrepancy due to difference in age.

*Strain*.—In mice the existence of strains with an incidence of mammary cancer varying from zero to a high percentage has long been known and the demonstration of the presence or absence of the milk agent in such strains indicates the reason for the varying incidence. In no other species have such nearly homozygous strains been developed, but in quite a number of laboratories both in this country and the United States groups of rats have been inbred over a considerable number of years, to such an extent that though the breeding methods used have not been of the standard used in mice, yet it may be reasonably assumed that the animals within each group are closely related genetically and that one group differs from another at least as much as do the different races of mankind. Such an assumption may help to explain the varying incidence of mammary tumours obtained by different workers and emphasizes the necessity for an investigation of the incidence of such tumours in different strains of rats under strictly comparable experimental conditions.

### EXPLANATION OF PLATES.

FIG. 7.—Section of breast from oestradiol treated rat showing precancerous proliferation within the ducts. × 128.

FIG. 8.—Section of breast from oestradiol treated rat showing papillary carcinoma. × 128.

FIG. 9.—Breast from oestradiol treated rat showing spheroidal cell carcinoma with squamous metaplasia. × 128.

FIG. 10.—Breast from stilboestrol treated rat showing adenocarcinoma. × 128.

FIG. 11.—Section of breast tumour from control rate (hooded). Pleomorphic celled sarcoma. × 128.

FIG. 12.—Section of fibro-adenomatous tumour from breast of control rat. × 128.

FIG. 13.—Section showing carcinoma of the ovary from a stilboestrol treated rate. × 128.

FIG. 14.—Section showing carcinoma of uterus in a stilboestrol treated rat. × 128.

7

8

9

10

MacKenzie.

11

12

13

14

MacKenzie.

The results obtained in the experimental part of this paper indicate that there is indeed a considerable difference in susceptibility among different strains of rats, and from these results and those of others one can divide them provisionally into high, medium and low cancer strains. Table III shows the strains, so far tested, assigned to each group.

TABLE III.—*Showing Susceptibility to the Development of Mammary Cancer by Oestrogen Stimulation among Strains of Rats so far Tested.*

| High cancer strains. | Per cent. | | Medium cancer trains. | Per cent. | | Low cancer strains. | Per cent. |
|---|---|---|---|---|---|---|---|
| Hooded rats (Unknown ancestry) (Noble *et al.*, 1940) | 55 | . | Inbred albino rats (Geschickter and Byrnes, 1942) | 36·4 | . | Inbred Sherman rats (Eisen, 1942) | 1·5 |
| Long-Evans hooded rats (Nelson, 1944) | 66 | . | A × C line 9935 (Dunning *et al.*, 1947) | 33 | . | Copenhagen line 2331 (Dunning *et al.*, 1947) | 0 |
| | | | Newcastle hooded rats (MacKenzie) | 24 | . | Fischer line 344 (Dunning *et al.*, 1947) | 6·3 |
| | | | WAG rats (MacKenzie) | 21·7 | . | Cancer Hospital rats (MacKenzie) | 5 |

It should be stressed however that these figures are not strictly comparable, in that (*a*) the ages of the animals at the beginning of the various experiments varied and (*b*) the oestrogen used, its dosage and method of administration also varied. Further work requires to be done to clarify and extend this observation.

*Oestrogenic substances employed.*

A considerable number of oestrogens have been used by various workers and Table IV summarises the results obtained. It should be emphasized, however that the experimental conditions under which they were used differed widely, and the fact that no tumours were obtained with any particular substance does not necessarily imply that it does not possess tumour-evoking properties. Until they have all been tested under identical conditions of dosage and time, using a strain of rats of known susceptibility, no definite conclusions can be drawn. In the present work tumours were obtained with both oestradiol and its dipropionate derivative, but the experimental conditions used were not such as to warrant any conclusions being drawn as to their relative effectiveness. Stilboestrol, on the other hand, while evoking the usual cystadenosis and occasional benign tumour, produced only 3 malignant tumours among 48 animals in these experiments. In view of the positive results obtained by others (Geschickter and Byrnes, 1942 ; Nelson, 1944 ; Dunning, Curtis and Segaloff, 1947) there must be some reason for this, and a review of the methods used by these workers suggests that this may be a matter of dosage. The amount used in the present experiments was a total of 10 mg. implanted intramuscularly as 2 pellets, whereas both Nelson and Dunning and his co-workers used a larger quantity. The latter workers, recognising that stilboestrol alone is absorbed fairly rapidly, incorporated it in pellets mixed with cholesterol in the proportion of 1 to 4. This greatly slowed the rate of absorption of the stilboestrol, and only after they had done so were they able to obtain breast tumours in their experimental animals. Geschickter and Byrnes (1942), on the other hand, were regularly able to obtain breast tumours with no more than 10 mg. of stilboestrol implanted subcutaneously as pellets. However, the general appearance of the stilboestrol implanted animals in the present series (particularly their weight and the relatively small increase in size of their pituitaries) strongly suggested that the dosage was inadequate, and further work utilizing in particular the technique of Dunning, Curtis and Segaloff (1947), is required to clarify this point.

TABLE IV.—*List of Oestrogens Employed Experimentally in Rats. (Summarized from Hartwell,* 1951)

| Oestrogen. | Breast tumours. |
|---|---|
| Oestradiol | Tumours obtained (Geschickter and Byrnes, 1942; MacKenzie). |
| „ benzoate | Tumours obtained (Mark and Biskind, 1941; Geschickter and Byrnes, 1942). |
| „ dipalmitate | No tumours. |
| „ dipropionate | Tumours obtained (Eisen, 1942; Geschickter and Byrnes, 1942; MacKenzie). |
| Oestradiol-17-caprylate | No tumours. |
| Oestradiol di-n-butyrate | „ „ |
| „ di-caprate | „ „ |
| „ di-n-hexanoate | „ „ |
| „ di-isobutyrate | „ „ |
| „ di-n-octanoate | „ „ |
| Oestradiol-3-benzoate-17-acetate | „ „ |
| Oestradiol-3-benzoate-17-butyrate | „ „ |
| Oestradiol-3-benzoate-17-propionate | „ „ |
| Oestradiol-3-benzoate-17n-valerate | „ „ |
| Oestradiol-3n-butyrate-17-benzoate | „ „ |
| Oestriol | „ „ |
| Oestrone | Tumours obtained (McEuen, 1938; Geschickter, 1939*a*, 1939*b*; Geschickter and Byrnes, 1942; Noble *et* al., 1940; Nelson, 1944). |
| Oestrone benzoate | Tumours obtained (Chamorro, 1943). |
| Oestrone-n-hexanoate | No tumours. |
| Oestrone-n-octanoate | No tumours. |
| Stilboestrol (4,4′-dihydroxy-αβ-diethyl-stilbene) | Tumours obtained (Geschickter and Byrnes, 1942; Nelson, 1944; Dunning *et al.*, 1947; MacKenzie). |
| Stilboestrol mono-methyl ether }<br>„ di-methyl ether } | Tumours obtained (Geschickter and Byrnes, 1942). |

The substances which have so far been successfully used in the production of breast tumours are oestradiol and its benzoate and dipropionate derivatives—oestrone and oestrone benzoate—and stilboestrol and its mono- and di-methyl derivatives. Of these Geschickter and Byrnes (1942) regarded oestradiol benzoate as the most effective.

*Dosage.*—Table V gives the dosages used in the various successful experiments, and it will be seen that they vary considerably. However, from a study of the various papers it would seem probable that a minimum of 10 mg. in pellet form of an active oestrogen is required to produce mammary cancer in rats and this agrees substantially with the conclusions reached by Geschickter and Byrnes (1942). In the case of a substance such as stilboestrol, which is rapidly absorbed, some agent like cholesterol, which retards absorption, should be mixed with it before the pellets are made.

*Method of Administration.*—Two methods have been used: (*a*) subcutaneous injection of the pure substance dissolved in oil and (*b*) subcutaneous or intramuscular implanation of pellets of the pure substance or of the pure substance mixed with cholesterol. Both of these methods have been successful in producing

TABLE V.—*Dosage of Oestrogens Used by Various Workers to Produce Tumours in Rats.*

| Author. | Strain of rat. | Oestrogens employed. | Method of administration and age at beginning of experiment. | Number with mammary cancer. | Age at first appearance of cancer (days). |
|---|---|---|---|---|---|
| McEuen (1938) | Hooded rats of unknown ancestry (ovariectomized) | Oestrone in corn oil 30 μg. daily | Subcutaneously; 106 days | 1/22 | 624 |
| Noble *et al.* (1940) | Same strain (intact) | Oestrone 4–11 mg. pellets | Subcutaneous implantation; 5–7 days | 28/49 | 226 |
| Geschickter and Byrnes (1942) | Inbred albino | Oestrone; oestradiol; oestradiol dipropionate and benzoate; stilboestrol | Subcutaneously by injection or implantation of pellets (dosage varied considerably); 2 days to 9 months | 202/555 | 125–170 (using oestradiol benzoate) |
| Eisen (1942) | Inbred Sherman | Oestradiol dipropionate, 2×5 mg. in paraffin wax | Subcutaneously as pellets (a few only) or subcutaneously, intra-muscularly or intraperitoneally in liquid paraffin wax; 3–5 weeks | 2/134 | 376 |
| Chamorro (1943) | Unstated | Oestrone benzoate in oil, 50–100 μg. | Subcutaneously by injection; 3 weeks | 6/10 | 609 |
| Dunning *et al.* (1947) | A × C line 9935; Fischer line 344; Copenhagen line 2331 | Stilboestrol; (*a*) 15–25 mg. pellet repeated, unsuccessful; (*b*) 4–15 mg. in Cholesterol (1 to 4) successful | Subcutaneously as pellets; 3–4 months | 39/117<br>6/95<br>0/108 | 320 |
| Nelson (1944) | Long-Evans hooded | Oestrone, 3 to 6 × 10 mg. pellets; stilboestrol by injection, 50μ daily, 1–4 × 10 mg. pellets | Subcutaneously by injection or implantation of pellets; 60–80 days | 68/103 | 130 (using oestrone) |
| MacKenzie | WAG (albino)<br>Newcastle (hooded); Cancer Hospital (albino) | Oestradiol, oestradiol dipropionate; stilboestrol; 2 × 6 mg. pellets | Intramuscular implantation of pellets; 28 days | 10/46<br>8/33<br>3/61 | 230 (using oestradiol) |

tumours. The difference between them lies in the method of absorption, which in the one case is discontinuous while in the other there is a continuous gradual absorption of the active substance, with the result that a considerably smaller total dose is required to produce the desired effect. Geschickter and Byrnes (1942) found that only one third to one half of the amount given by daily injection was required to produce mammary cancer if given in the form of pellet implants; in addition, the great saving in labour need not be stressed. With regard to the vehicle in which the active substance is given, when the injection technique is used it is dissolved in some oil, such as corn oil or olive oil. Pellets are made of the pure substance, but, as already noted, Dunning Curtis and Segaloff (1947) have demonstrated the value of mixing it with cholesterol when stilboestrol is being used. Eisen (1942), however, used oestradiol dipropionate dissolved in liquified paraffin which, when cooled in the body, formed a solid wax pellet from which the active substance was gradually absorbed. However, these pellets appeared to evoke a marked acellular connective tissue reaction around them and it is possible that the rate of absorbtion was much reduced, thus accounting for the low incidence of tumours which he obtained.

*Duration of stimulation.*—From Table V it will be seen that the time from the onset of oestrogenic stimulation to the appearance of palpable nodules of mammary cancer varies considerably. This may well be a product of three variables: (*a*) strain of rat, (*b*) type of oestrogen, and (*c*) dosage; and it is difficult to know what the relative importance of each factor may be. From the figures, however, it appears that palpable nodules are unlikely to appear in less than 120 days, though tumours may be discovered microscopically before that. Therefore any experiment must be continued either (*a*) until the appearance of a tumour or (*b*) until the death of the animal. In the present experiments the earliest tumour was noted about the 230th day after the beginning of oestrogenic stimulation, but others developed much later than this. Geschickter and Byrnes (1942) found that the time of appearance of a tumour depended largely on the dosage of oestrogen given, the larger the dose within a given limit (200$\mu$g. daily in the case of oestrone) the earlier the tumours appeared. Also they state that the older the animal is at the onset of stimulation the larger the dose must be to cause the the appearance of cancer. Amounts greater than the optimum did not decrease the time of appearance of the tumours, nor increase the number of animals with tumours.

### SUMMARY.

1. Female rats of three different strains were implanted with oestradiol, oestradiol dipropionate, or stilboestrol, and kept under observation till they died.

2. Out of a total of 140 female animals, 21 developed malignant mammary tumours, some of them showing multiple tumours.

3. Four animals developed malignant tumours of the ovary or uterus.

4. The experimental conditions necessary to the production of malignant tumours of the breast in rats are discussed.

### REFERENCES.

ANDERVONT, H. B.—(1945) A Symposium on Mammary Tumours in Mice. *Amer. Ass. Adv. Sci.*, p. 130.

CHAMORRO, A.—(1943) *C. R. Soc. Biol. Paris*, **137**, 325.
DUNNING, W. F., CURTIS, M. R. AND SEGALOFF, A.—(1947) *Cancer Res.*, **7**, 511.
EISEN, M. J.—(1942) *Ibid.*, **2**, 632.
GESCHICKTER, C. F.—(1939*a*) *Science*, **89**, 35. (1939*b*) *Radiology* **33**, 439,
*Idem* AND BYRNES, E. W.—(1942) *Arch. Path.*, **33**, 334.
HARTWELL, J. L.—(1951) 'Survey of Compounds which have been tested for carcinogenic activity.' U.S. Public Health Service, Publication No. 149.
MCEUEN, C. S.—(1938) *Amer. J. Cancer*, **34**, 184.
MARK, J. AND BISKIND, J. R.—(1941) *Endocrinology*, **28**, 465.
NELSON, W. O.—(1944) *Yale J. Biol. Med.*, **17**, 217.
NOBLE, R. L., MCEUEN, C. S. AND COLLIP, J. B.—(1940) *Canad. med. Ass. J.*, **42**, 413.